# Metagenome-assembled genomes from urban pigeon feces in Istanbul, Türkiye

**DOI:** 10.1128/mra.01405-25

**Published:** 2026-04-20

**Authors:** İrem Kocakahya, Gül Şahin, Erdem Büyükkahraman, Muzaffer Arıkan

**Affiliations:** 1 Biotechnology Division, Department of Biology, Faculty of Science, Istanbul University 369915, Istanbul, Turkey; Montana State University, Bozeman, Montana, USA

**Keywords:** pigeon, metagenomics, fecal microbiome, DNA sequencing, bacterial genome

## Abstract

We report herein about 101 metagenome-assembled genomes (MAGs) obtained from pigeon fecal samples collected in 2025 from the Beyazıt, Kadıköy, and Beşiktaş squares of Istanbul. The MAGs were predominantly composed of members of the phyla *Firmicutes*, *Actinobacteria*, and *Proteobacteria*, with a lower representation of *Campylobacterota* and *Patescibacteriota*.

## ANNOUNCEMENT

Pigeons (*Columba livia domestica*) are ubiquitous in urban ecosystems and serve as important indicators of environmental microbial dynamics. Their feces can act as reservoirs and potential vectors for the dissemination of antibiotic resistance genes (ARGs), posing as a significant public health concern in densely populated cities. In this study, a total of 75 pigeon fecal samples were collected from three major public squares in Istanbul: Beyazıt (41.010266 N, 28.964197 E), Kadıköy (40.992596 N, 29.023987 E), and Beşiktaş (41.042404 N, 29.007358 E). Samples were aseptically transferred to sterile containers, transported on ice, and stored at −20°C until further processing. Total DNA was extracted using the Qiagen DNeasy PowerSoil Pro Kit as previously described (1). Five randomly selected DNA samples from each square were quantified using the Qubit dsDNA HS assay and pooled at equal DNA concentrations, resulting in a total of 15 pooled samples for sequencing. Sequencing libraries were prepared from 100 ng of purified DNA using the Illumina DNA Prep Kit and sequenced on the Illumina NovaSeq 6000 platform using paired-end 2 × 150 bp reads following the manufacturer’s default protocol. Library construction failed for one sample, resulting in 14 metagenomic samples.

Unless otherwise specified, all bioinformatics tools were run using their default parameters recommended by the developers. Raw sequencing reads were subjected to quality control using FastQC (v0.12.1) (2), and then processed with Trimmomatic (v0.39) (3) to remove adapters and low-quality bases using the parameters: ILLUMINACLIP:TruSeq3-PE.fa:2:30:10, LEADING:3, TRAILING:3, SLIDINGWINDOW:4:20, and MINLEN:36. The cleaned paired-end reads were mapped to the *Columba livia* reference genome using Minimap2 (v2.24) (4), and unmapped reads were retained using Samtools (v1.18) (5) to exclude host-derived sequences. Metagenomic assemblies were generated using metaSPAdes (v3.15.5) (6), and binning was performed in three stages using MetaBAT2 (7), MaxBin 2.0 (8), and CONCOCT (9), after which the resulting bins were integrated using DAS Tool (10) to produce a consensus set. Quality evaluation was conducted with CheckM2 (v1.0) (11), with bins meeting ≥90% completeness and ≤5% contamination retained as high-quality MAGs based on MIMAG recommendations (12). The final high-quality MAGs were classified taxonomically using the Genome Taxonomy Database (GTDB) (Release 10-RS226) (13).

The sequencing effort generated a total of 75 Gbp of data, averaging 5.4 Gbp per sample (approximately 18 million read pairs or 36 million reads per sample), enabling high-resolution metagenomic characterization of the urban pigeon fecal microbiome. Quality control analyses showed that, on average, 90% of bases per sample had a quality score of Q30 or higher. Assembly and binning yielded 101 high-quality MAGs, with a median completeness of 95.46% and a median contamination of 0.96% (see Table S1 at https://doi.org/10.6084/m9.figshare.30801050). GTDB-based taxonomic assignments revealed *Firmicutes*, *Actinobacteriota*, and *Proteobacteriota* as the dominant phyla, with additional representation from *Campylobacterota* and *Patescibacteriota* (see Table S1 at https://doi.org/10.6084/m9.figshare.30801050).

## Data Availability

The shotgun sequence data have been deposited at the NCBI Sequence Read Archive under BioProject accession number PRJNA1375659. The recovered MAGs and the spreadsheet detailing characteristics of these metagenome-assembled genomes can be found on Figshare at https://doi.org/10.6084/m9.figshare.30801050.
